# An allocentric exception confirms an egocentric rule: a comment on Taghizadeh and Gail (2014)

**DOI:** 10.3389/fnhum.2014.00942

**Published:** 2014-12-02

**Authors:** Paul Dassonville, Benjamin D. Lester, Scott A. Reed

**Affiliations:** ^1^Department of Psychology and Institute of Neuroscience, University of OregonEugene, OR, USA; ^2^Department of Neurology, University of Iowa Hospitals and ClinicsIowa City, IA, USA

**Keywords:** visual perception, illusion, egocentric frame of reference, allocentric frame of reference, motor control

When a visual cue is presented in the context of a large rectangle shifted laterally from an observer's midline, its location is perceived to be shifted in the opposite direction (a phenomenon known as the induced Roelofs effect, a variant of an illusion first discovered by Roelofs, 1936). However, movements made immediately to the cue are accurate (Bridgeman et al., 1997; Dassonville and Bala, 2004a). We have shown evidence that the perceptual effect of the illusion is brought about by a distortion of the observer's egocentric reference frame—the offset rectangle attracts the observer's subjective straight-ahead (SSA), causing the cue to appear to be shifted in the opposite direction. However, if an action aimed at the cue is then guided within this same distorted egocentric reference frame, the error of motor guidance will cancel with the error of perceptual encoding, allowing the movement to be accurate (Dassonville and Bala, 2004a,b; Dassonville et al., 2004). We have begun to refer to this cancelation of errors, which allows for accurate actions in spite of the illusion, as the Two-Wrongs model, since, according to the model, two wrongs *do* make a right (Dassonville and Reed, under review). However, in a recent exploration of the induced Roelofs effect (IRE) on allocentrically-guided movements, Taghizadeh and Gail (2014) purport to show evidence against the Two-Wrongs model. A closer examination, though, reveals flaws in their assumptions, leading us to conclude that the Two-Wrongs model is, in fact, completely supported by their data. Here, we critically assess each of the three pieces of evidence used to argue against the Two-Wrongs model.

In Experiment II of Taghizadeh and Gail (2014), participants were first shown a reference array of possible cue locations (positioned to the left or right of the mid-sagittal plane), followed by a cue presented within a Roelofs-inducing rectangle. Participants were required to note the location of the cue within the previously-presented reference array, and then point to the same allocentric location in a subsequent decision array. In certain critical trial types, the authors found errors that were in the opposite direction of those typically seen with the IRE. Based on their assumption that a distortion of the egocentric reference frame could only cause an error in the direction opposite that of the inducing rectangle, the authors concluded that the illusion must not be caused by such a distortion. However, their assumption is patently incorrect, since they fail to account for the initial influence of the *reference array itself* on the SSA. After all, there is nothing special about the typical Roelofs-inducing rectangle, other than its lateralized location—any lateralized stimulus would be expected to induce a similar distortion (e.g., Wapner et al., 1953; Walter and Dassonville, 2006; Lester and Dassonville, 2013), although its magnitude might be modulated by salience, attention, etc. (Lester and Dassonville, 2011). In the paradigm of Taghizadeh and Gail, when the reference array appears in the left hemifield, its presence would cause the SSA to be pulled to the left (Figure 1A), and the perceived location of the array would be encoded within this distorted reference frame. When the large inducing rectangle is later presented, it would exert its own influence on the SSA, but, since it is not as lateralized as the reference array, it would drag the SSA (and the memory of the reference array) *rightward* from where it had been at the time of the reference array presentation (Figure 1B). Accordingly, a cue presented at the center of the reference array would be reported as being to the left of center in the remembered array, even though the absolute position of the inducing rectangle was to the participant's left. Thus, an account of the IRE based on a distorted egocentric reference frame fully predicts that the resulting errors will depend on the relative displacement of the SSA between the occurrence of the reference array and cue/rectangle, not the rectangle's absolute position in space.

**Figure 1:**
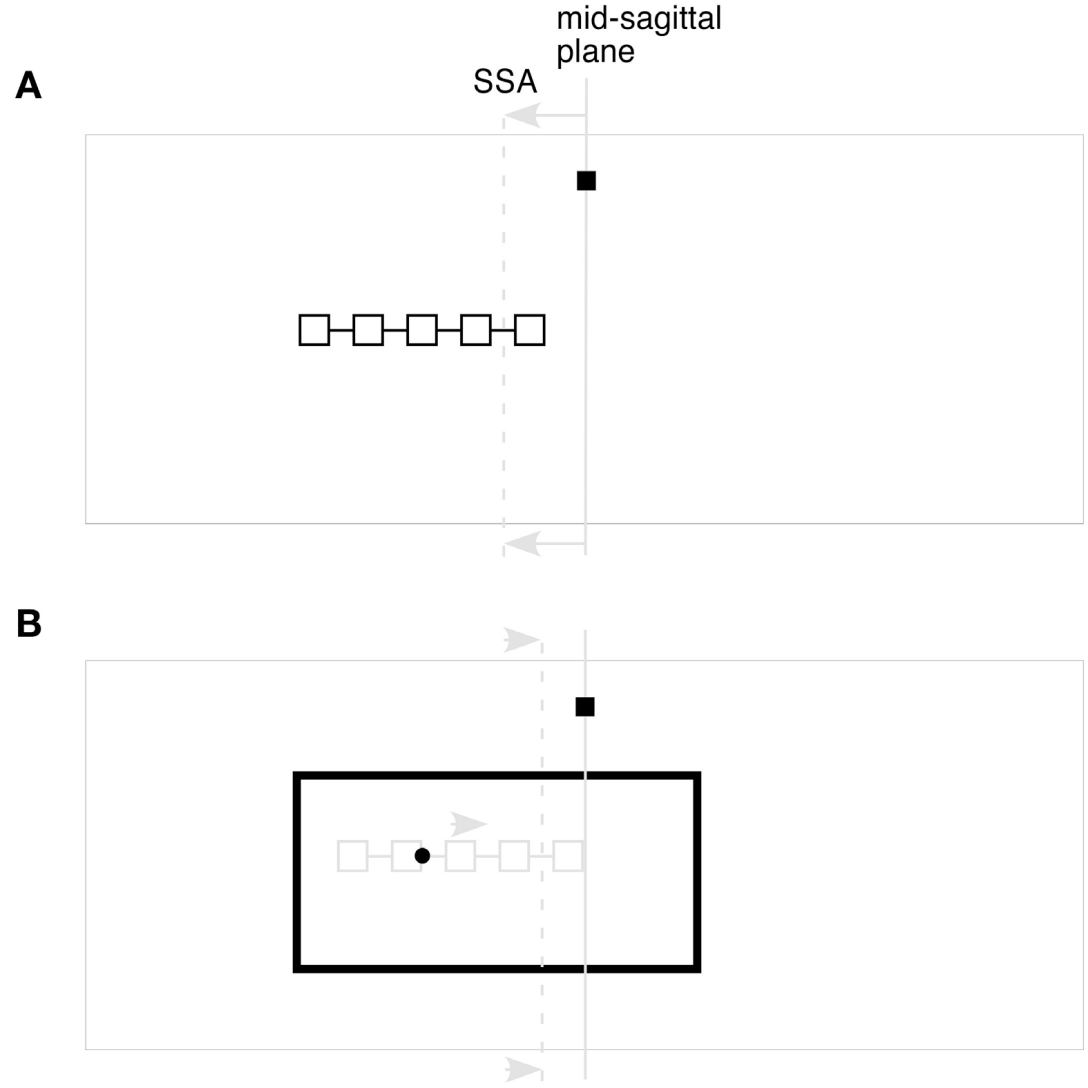
**Distortions of the SSA in Experiment II of Taghizadeh and Gail (2014), according to the predictions of the Two-Wrongs model of visual processing. (A)** The initial step of stimulus presentation, showing the central fixation point (small square), and a reference array shifted to the left of the participant's mid-sagittal plane (solid gray line). Like the offset rectangle in the typical IRE, the offset reference array would serve to attract the participant's SSA (dashed gray line). **(B)** The subsequent appearance of the cue and Roelofs-inducing rectangle, after the reference array has been extinguished. In spite of the inducing rectangle's bias toward the left hemifield, its center of gravity is not as lateralized as that of the earlier reference array. This would cause the SSA to move rightward, pulling the memory of the reference array with it, and causing the cue to be mislocalized toward the left end of the remembered reference array. Although the image shows the SSA as moving directly between the distorted positions caused by the sequential presentation of the reference array and the later Roelofs-inducing rectangle, this change in the SSA need not be direct (for example, the SSA may drift back toward the objective midline during the delay period between reference array and inducing rectangle presentations, only to be pulled leftward again when the inducing rectangle is presented; see Dassonville and Bala, 2004a). Importantly, the direction and magnitude of the IRE would depend only on the relative locations of the distorted SSA during reference array and target presentations, regardless of its possible meanderings between those events.

The authors also argue that the illusion's effect on immediate movements in their paradigm provides evidence against the Two-Wrongs model. However, the model specifically predicts that accurate movements will occur *only* when they are aimed at the *egocentric* location of the cue (Dassonville and Bala, 2004a; Dassonville et al., 2004). In contrast, the task of Taghizadeh and Gail required participants to guide their response to the *allocentric* location of the cue, and therefore the cancelation of errors described by the Two-Wrongs model would not occur. Given this, the data of Taghizadeh and Gail do not provide evidence against the Two-Wrongs model, but instead confirm the model's prediction that accurate movements will only occur in the face of the IRE when they are aimed at the cue's location within an egocentric reference frame.

Finally, the authors argue against an egocentric account of the IRE by pointing to their analysis that seems to suggest that the presence or absence of a fixation point has no effect on the illusion, claiming that a fixation point should provide an anchor that would stabilize the reference frame and eliminate the illusion. We agree that a fixation point *could* have a stabilizing effect, but there is no *a priori* reason to expect that it *must*, especially since the illusion is modulated by the salience of, and amount of attention directed toward, the inducing stimulus (Lester and Dassonville, 2011). Thus, it could have been anticipated that the effects of the large, salient inducing rectangle would largely overcome any stabilizing effects of the small fixation point or dimly lit laboratory.

Contrary to the conclusions of Taghizadeh and Gail (2014), their results are fully compatible with the hypothesis that the IRE is caused by a distortion in the observer's egocentric reference frame. Moreover, they provide confirmatory evidence for the Two-Wrongs model and its prediction that movements made in the context of the illusion will be accurate only when they are guided within the same distorted egocentric frame that is used to encode the cue's location (Dassonville and Bala, 2004a; Dassonville et al., 2004; Dassonville and Reed, under review).

## Conflict of interest statement

The authors declare that the research was conducted in the absence of any commercial or financial relationships that could be construed as a potential conflict of interest.

## References

[B1] Bridgeman B. Peery S. Anand S. (1997). Interaction of cognitive and sensorimotor maps of visual space. Percept. Psychophys. 59, 456–469. 10.3758/BF03211912 9136275

[B2] Dassonville P. Bala J. K. (2004a). Action, perception and the Roelofs effect: a mere illusion of dissociation. PLoS Biol. 2:1936–1945. 10.1371/journal.pbio.0020364 15510224 PMC524248

[B3] Dassonville P. Bala J. K. (2004b). Are the original Roelofs effect and the induced Roelofs effect confounded by the same expansion of remembered space? Vis. Res. 44, 1025–1029. 10.1016/j.visres.2003.10.018 15031095

[B4] Dassonville P. Bridgeman B. Bala J. K. Thiem P. Sampanes A. (2004). The induced Roelofs effect: two visual system of the shift of a single reference frame? Vis. Res. 44, 603–611. 10.1016/j.visres.2003.10.017 14693187

[B5] Lester B. D. Dassonville P. (2011). Attentional control settings modulate susceptibility to the induced Roelofs effect. Atten. Percept. Psychophys. 73, 1398–1406. 10.3758/s13414-011-0123-9 21479725 PMC3546118

[B6] Lester B. D. Dassonville P. (2013). Shifts of visuospatial attention do not cause the spatial distortions of the Roelofs effect. J. Vis. 13, 1–15. 10.1167/13.12.4 24105425

[B7] Roelofs C. O. (1936). Die optische Lokalisation [Visual localization]. Archiv für Augenheilkunde 109, 395–415.

[B8] Taghizadeh B. Gail A. (2014). Spatial task context makes short-latency reaches prone to induced Roelofs illusion. Front. Hum. Neurosci. 8:673. 10.3389/fnhum.2014.00673 25221500 PMC4148936

[B9] Walter E. Dassonville P. (2006). Fragments of the Roelofs effect: a bottom-up effect equal to the sum of its parts. Percept. Psychophys. 68, 1243–1253. 10.3758/BF03193724 17378411

[B10] Wapner S. Werner H. Bruell J. H. Goldstein A. G. (1953). Experiments on sensory-tonic field theory of perception: VII. Effect of asymmetrical extent and starting positions of figures on the visual apparent median plane. J. Exp. Psychol. 46, 300–307. 13109130 10.1037/h0056203

